# Ultrasound-Guided Jugular Vein Access for Inferior Petrosal Sinus Sampling: A Safe and Feasible Technique

**DOI:** 10.7759/cureus.96167

**Published:** 2025-11-05

**Authors:** Diego Páez-Granda, Stefany Baquero, Jorge Luis Salazar Vega, Gloria Estévez

**Affiliations:** 1 Neuroradiology Section, Hospital Metropolitano, Quito, ECU; 2 Neurointerventional Radiology Department, Hospital de Especialidades Eugenio Espejo, Quito, ECU; 3 Endocrinology, Diabetes and Metabolism Department, Hospital de Especialidades Eugenio Espejo, Quito, ECU; 4 Radiology Department, Hospital de Especialidades Eugenio Espejo, Quito, ECU

**Keywords:** inferior petrosal sinus sampling, interventional radiology, jugular puncture, pituitary cushing’s disease, ultrasound-guided

## Abstract

Pituitary Cushing’s disease (CD) results from excessive adrenocorticotropic hormone (ACTH) secretion, usually due to a pituitary adenoma. This report describes the diagnostic approach and management of a complex case of CD in a patient with multiple comorbidities, highlighting a hybrid technique for inferior petrosal sinus sampling (IPSS) when standard access fails.

A woman with poorly controlled diabetes, obesity, chronic kidney disease (CKD), and hypertension presented with suspected Cushing’s syndrome. Despite normal urinary free cortisol (UFC) levels (likely influenced by renal dysfunction), clinical suspicion prompted further testing, which revealed an inverted cortisol rhythm and lack of suppression on low-dose dexamethasone. High-dose suppression indicated a pituitary source. MRI findings were inconclusive. To confirm the diagnosis, bilateral IPSS was attempted. Right petrosal sinus catheterization via femoral access was successful; however, left-sided access failed. An alternative, ultrasound-guided direct left internal jugular puncture was performed, allowing complete sampling. A central-to-peripheral ACTH gradient >2 at baseline and >3 after desmopressin confirmed a pituitary source. The patient subsequently underwent successful transsphenoidal resection, achieving postoperative biochemical remission.

IPSS remains the gold standard for distinguishing central from ectopic ACTH production. While bilateral femoral access is standard, anatomical variants may necessitate alternative routes. This case demonstrates the feasibility and safety of combining femoral and direct jugular access to complete IPSS when conventional approaches are limited.

This is the first reported case of IPSS performed using a hybrid right femoral and left ultrasound-guided jugular approach, offering a practical alternative when femoral access is not feasible and reinforcing the diagnostic value of IPSS in challenging cases.

## Introduction

Pituitary Cushing’s disease (CD) is caused by excessive secretion of adrenocorticotropic hormone (ACTH), typically due to a pituitary adenoma. It represents the most common cause of endogenous Cushing’s syndrome, accounting for approximately 70% of ACTH-dependent cases [1,2]. The diagnostic approach often requires dynamic hormonal testing and neuroimaging; however, distinguishing pituitary from ectopic ACTH secretion remains a clinical challenge [3].

Inferior petrosal sinus sampling (IPSS), first described by Oldfield EH and Doppman JL in 1977, is considered the gold standard for confirming a pituitary origin when biochemical and imaging findings are inconclusive [4-6]. Bilateral catheterization via femoral venous access is the usual approach, guided by digital subtraction angiography (DSA) [4,5]. However, anatomical variants, thrombosis, and technical difficulties can impede standard catheterization, necessitating alternative strategies such as direct ultrasound-guided internal jugular puncture [7].

This report presents a patient with multiple comorbidities and suspected CD in whom a hybrid IPSS approach was successfully performed after failed standard access.

## Case presentation

A female patient with a history of poorly controlled diabetes, obesity, chronic kidney disease (CKD), and hypertension was admitted with suspected Cushing’s syndrome. Initial evaluation revealed normal urinary free cortisol (UFC), likely underestimated due to renal dysfunction. Because of high clinical suspicion, circadian cortisol rhythm was assessed, showing inversion with higher evening than morning levels, supporting hypercortisolism.

A low-dose dexamethasone suppression test (LDDST; 1 mg) failed to suppress cortisol, confirming endogenous hypercortisolism. A high-dose dexamethasone suppression test (HDDST; 8 mg) demonstrated 80% cortisol suppression, suggesting a pituitary source of ACTH overproduction.

**Figure 1:**
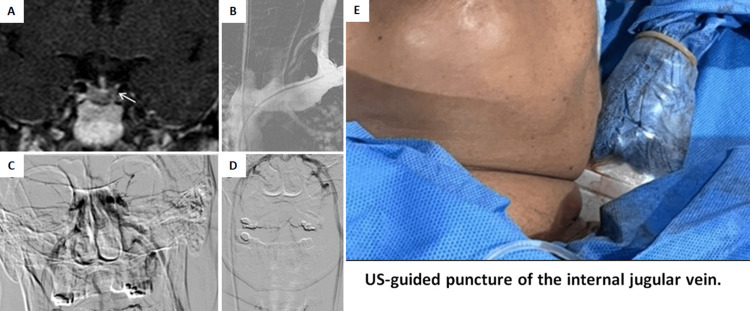
(A) Contrast-enhanced pituitary MRI showing a hypointense nodule in the left half of the gland, which was inconclusive; (B) right internal jugular vein access achieved, while left jugular access was not possible via this route; (C and D) dual inferior petrosal sinus catheterization with right-sided access via the femoral vein and left-sided access via direct jugular puncture; (E) ultrasound-guided placement of the venous sheath.

Pituitary MRI revealed a poorly defined hypointense nodular area, inconclusive for microadenoma (Figure 1A). To confirm the central origin, bilateral inferior petrosal sinus sampling (IPSS) was performed (Figures 1B-1E).

Initial access was established via the bilateral femoral veins with placement of 5 Fr introducer sheaths in both. Due to anatomical complexity and inability to access the left internal jugular vein via the femoral route, a direct ultrasound-guided left jugular puncture was performed. A separate 5 Fr introducer sheath was placed directly into the left internal jugular vein under ultrasound guidance (US guidance). Catheterization was performed using 5 Fr vertebral diagnostic catheters, facilitated by a micro-guidewire.

Correct positioning within the petrosal sinuses was subsequently confirmed by contrast injection. The results demonstrated accurate catheter placement in the inferior petrosal sinuses (adequate prolactin levels), with an ACTH central-to-peripheral gradient greater than 2 at baseline and greater than 3 after desmopressin, thus confirming a pituitary source for the pathology (Tables 1-2).

**Table 1:** Prolactin concentrations obtained via inferior petrosal sinus sampling at baseline. IPS: Inferior Petrosal Sinus.

Peripheral	Right IPS	Left IPS
16.5 ng/mL	41.2 ng/mL	63.7 ng/mL

**Table 2:** ACTH concentrations obtained via inferior petrosal sinus sampling at baseline and at 5 and 10 minutes after desmopressin stimulation. IPS: Inferior Petrosal Sinus; ACTH: Adrenocorticotropic hormone.

Time Point	Peripheral	Right IPS	Left IPS
Basal	27.5 pg/mL	77.1 pg/mL	106 pg/mL
Desmopressin 5 min	28.3 pg/mL	168 pg/mL	221 pg/mL
Desmopressin 10 min	27.9 pg/mL	32 pg/mL	80 pg/mL

The patient underwent endonasal transsphenoidal resection of an ACTH-secreting pituitary microadenoma. Postoperatively, serum cortisol fell to <5 µg/dL, indicating secondary adrenal insufficiency, and physiologic glucocorticoid replacement was initiated. Urine output remained normal (no evidence of vasopressin deficiency), and steroid replacement was titrated without adrenal crisis.

## Discussion

Diagnostic considerations

CKD can lead to falsely normal UFC values due to impaired renal clearance of cortisol metabolites [8]. Therefore, alternative biochemical tests such as late-night serum cortisol or dexamethasone suppression are recommended in these patients [1,3]. The high-dose dexamethasone suppression observed here supported a pituitary origin, but confirmation by IPSS was critical given the inconclusive MRI findings.

Inferior petrosal sinus sampling

Since its introduction, IPSS has become the reference standard for distinguishing pituitary from ectopic ACTH production, with reported sensitivity and specificity of approximately 96% and 100%, respectively [4-6,9]. The test involves measuring ACTH gradients between central (petrosal) and peripheral samples, values ≥2 at baseline or ≥3 after corticotropin-releasing hormone (CRH) or desmopressin stimulation indicate a central source [5,9].

Desmopressin stimulation

Although CRH has traditionally been used, desmopressin is an effective and safe alternative that achieves comparable diagnostic accuracy [10]. In our case, desmopressin successfully elicited a diagnostic gradient, confirming the pituitary source.

Technical challenges and hybrid approach

Although the conventional IPSS technique uses bilateral femoral access, the procedure was originally performed via direct jugular puncture [2]. Variations in venous anatomy, hypoplasia, or catheterization failure may necessitate alternative routes. Direct ultrasound-guided jugular puncture offers an effective solution, minimizing procedural time and radiation exposure, and reducing the risk of complications such as cervical hematoma. Our case illustrates that combining femoral and direct jugular access allows complete bilateral sampling without compromising safety.

## Conclusions

This case demonstrates the feasibility and safety of a hybrid IPSS approach combining right femoral and ultrasound-guided direct left jugular access. This method enabled successful completion of bilateral sampling when standard femoral catheterization failed. The case reinforces IPSS as a critical diagnostic tool for confirming pituitary Cushing’s disease, even in technically challenging circumstances.
